# Laparoscopic hepatectomy for hepatic angiomyolipoma with preoperative diagnosis of other malignancy: a report of 2 cases

**DOI:** 10.1186/s40792-021-01125-7

**Published:** 2021-02-08

**Authors:** Yoh Asahi, Toshiya Kamiyama, Tatsuya Orimo, Shingo Shimada, Akihisa Nagatsu, Yuzuru Sakamoto, Chihiro Ishizuka, Kazuya Hamada, Hirofumi Kamachi, Emi Takakuwa, Tomoko Mitsuhashi, Akinobu Taketomi

**Affiliations:** 1 Department of Gastroenterological Surgery I, Hokkaido University Hospital, Kita-ku, Kita 15, Nishi 7, Sapporo, Hokkaido 060-8638 Japan; 2 Department of Surgical Pathology, Hokkaido University Hospital, Kita-ku, Kita 15, Nishi 7, Sapporo, Hokkaido 060-8638 Japan

**Keywords:** Laparoscopic hepatectomy, Hepatic angiomyolipoma

## Abstract

**Background:**

Hepatic angiomyolipoma (HAML) is a rare liver tumor, and hepatectomy is the only effective treatment. Due to the difficulty of correct diagnosis of HAML before surgery by image studies, more than 36.6% of reported HAMLs are misdiagnosed as other malignant liver tumors before surgery. As there are only few reported cases in which HAMLs were removed using laparoscopic hepatectomy, the effectiveness of laparoscopic hepatectomy for such HAMLs in which are diagnosed as other malignant liver tumor before surgery has not been reported.

Case presentation

Case 1: a 58-year-old female with a history of treatment for autoimmune hepatitis was preoperatively diagnosed with hepatocellular carcinoma (size: 20 mm) in segment 7 (S7) of the liver. The tumor was removed by laparoscopic partial resection and was diagnosed as a HAML through a pathological examination. The patient’s postoperative course was good, and she was recurrence-free at 37 months after the hepatectomy. Case 2: a 29-year-old female with a history of surgery for a right mature cystic teratoma was referred to our department to receive treatment for a growing 20-mm liver tumor with some calcification, which arose in S3 of the liver. A metastatic liver tumor derived from the mature cystic teratoma was suspected, and laparoscopic left lateral sectionectomy was performed. The liver tumor was diagnosed as a HAML after a pathological examination. The patient’s postoperative course was unremarkable, and more than 54 months have passed since the hepatectomy without any recurrence.

**Conclusions:**

Two cases in which HAMLs were preoperatively diagnosed as other malignant liver tumor were successfully removed by laparoscopic hepatectomy with a correct postoperative diagnosis. Laparoscopic hepatectomy for the present 2 cases of HAML seemed to be effective for providing a correct diagnosis after the curative removement of liver tumor with a smaller invasion compared to open hepatectomy, and for denying risk of dissemination of the malignant tumor by needle biopsy that had to be considered before ruling out malignant tumor.

## Introduction

Hepatic angiomyolipoma (HAML) is a rare type of liver tumor, consisting of thick-walled blood vessels, smooth muscle bundles, and adipose tissue in varying proportions [1]. Most AMLs arise in the kidneys, and the liver is the second-most common site; however, the precise incidence of HAML is unknown [2]. HAMLs mainly occur in young women with normal liver function, and most of them are sporadic. This is not the case for renal AML, in which > 50% of cases occur secondary to tuberous sclerosis [3].

Most HAMLs are considered to be benign, but there have been some case reports of HAMLs with malignant clinical courses, such as cases involving metastasis or recurrence [4, 5]. There have also been some cases reports about ruptured HAMLs [6] or giant HAMLs [7] with abdominal symptoms. These fatal or symptomatic cases of HAML suggest that some HAMLs need treatment. Unfortunately, due to the lack of reports or prospective trials relating to HAML, no treatment strategy for the disease has yet been established, and surgical resection with negative margins is the only effective treatment at present [2][2].

Furthermore, it can be difficult to preoperatively diagnose some HAMLs, and it was suggested that more than 36.6% of reported HAMLs are diagnosed as other malignant tumors before surgery, and diagnosed as HAML depending on pathological findings of resected specimens [2]. In cases of HAML that require liver resection, laparoscopic hepatectomy could be a selective choice for surgical method. However, there have only been two reports about HAMLs that were removed using a laparoscopic procedure [9][9], and neither of these cases involved HAML that were misdiagnosed as other malignant liver tumors before surgery. Herein, we report 2 cases of HAML in which was removed by laparoscopic procedure after the preoperative diagnosis of other malignant tumor and was finally diagnosed as HAML depending on pathological findings of resected specimens.

## Case 1

**Fig. 1:**
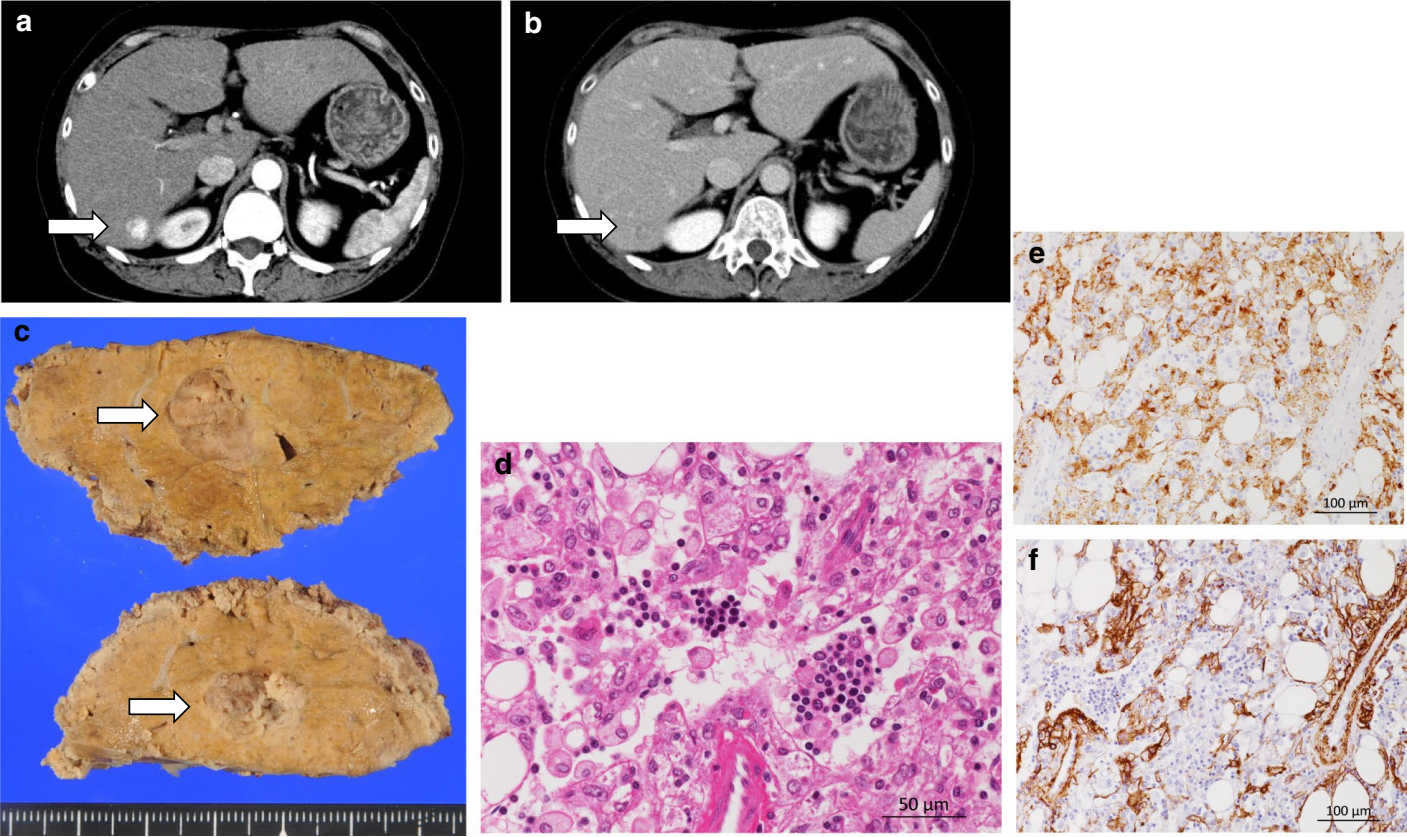
Images of case 1. **a** Arterial-phase contrast-enhanced CT image obtained in case 1. The tumor exhibited early enhancement (arrow: the tumor). **b** Delayed-phase contrast-enhanced CT image obtained in case 1. The tumor was not enhanced in the delayed phase (arrow: the tumor). **c** Resected specimen from case 1. The cut surface of the specimen contained a circumscribed solid tumor, which was light brownish in color (arrow: the tumor). **d** Hematoxylin and eosin (HE) staining of the tumor in a low-power field. The tumor cells exhibited sheet-like growth, were spindle-shaped to polygonal, and contained granular to clear cytoplasm. Adipocytes and blood vessels were present in the tumor. Extramedullary hematopoiesis was also seen (arrows). **e** HMB-45 staining of the tumor in a low-power field. The tumor was positive for HMB-45. **f** α-SMA staining of the tumor in a low-power field. The tumor was positive for α-SMA

A liver tumor was detected in a 58-year-old female during health screening. The patient had been diagnosed with hepatocellular carcinoma (HCC) at another hospital. She was referred to our department. She had a history of treatment for autoimmune hepatitis at the previous hospital. Her medical history also included Hashimoto’s disease and bronchial asthma. She was asymptomatic, and her general condition was good. Blood tests produced normal results regarding her complete blood count; coagulation function; renal function; and liver function. Tests for the hepatitis B surface antibody and hepatitis B core antibody were positive, suggesting a prior hepatitis B virus infection. A test for the hepatitis C virus antibody was negative. The patient’s levels of the tumor markers alpha-fetoprotein (AFP) and protein induced by vitamin K absence or antagonist-II (PIVKA-II) were within the normal ranges. Contrast-enhanced computed tomography (CT) showed a 20-mm tumor in segment 7 (S7) of the liver. The tumor exhibited hyper-enhancement in the arterial phase and washout in the portal venous and delayed phases. The tumor had a peripheral capsule (Fig. 1a, b). Laparoscopic partial resection of S7 and cholecystectomy were performed. The operation time was 4 h and 49 min, and the amount of intraoperative blood loss was 10 ml. The tumor was diagnosed as a HAML after a pathological examination (Fig. 1c–e). The patient’s postoperative course was unremarkable, and she was discharged on the 13th day after surgery. The tumor had not recurred at 37 months after surgery.

## Case 2

**Fig. 2:**
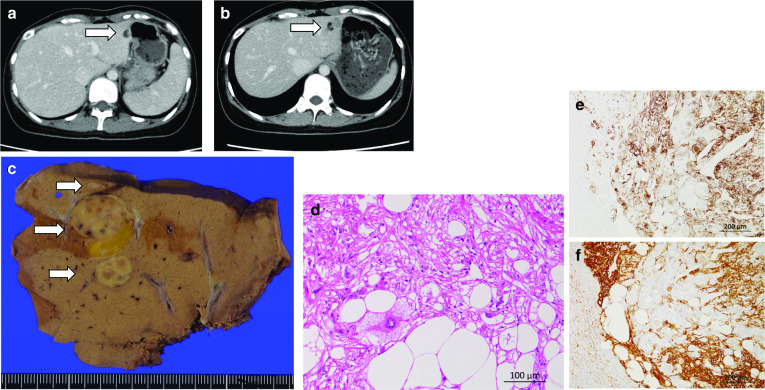
Images of case 2. **a** Contrast-enhanced CT image obtained in case 2 at the time of the first surgery (for the right mature cystic teratoma). The tumor measured 10 mm in diameter (arrow: the tumor). **b** Contrast-enhanced CT image obtained in case 2 at 2 years after the first surgery. The tumor measured 20 mm in diameter and possessed various components, including an adipose component (arrow: the tumor). **c** Resected specimen from case 2. The cut surface of the resected specimen contained a light brownish and yellow tumor, which measured 20 mm in diameter (arrow: the tumor). **d** HE staining of the tumor in a high-power field. The tumor consisted of a mixture of adipocytes, spindle-shaped cells, and epithelioid cells. Large atypical epithelioid cells with eosinophilic to clear cytoplasm were also identified. **e** HMB-45 staining of the tumor in a low-power field. The tumor was positive for HMB-45. f: α-SMA staining of the tumor in a low-power field. The tumor was positive for α-SMA

A 29-year-old female underwent left-sided adnexectomy for a left-sided yolk sac tumor and enucleation of a right ovarian mature cystic teratoma at our hospital’s Department of Gynecology. A small tumor was seen in S3 of the liver during a preoperative CT examination, which was suspected to be benign (Fig. 2a). The patient received adjuvant chemotherapy involving four courses of BEP (bleomycin, etoposide, and cisplatin) combination therapy after surgery. Two years after the first surgery, the liver tumor had grown to 20 mm in diameter, and the patient was referred to our department to have it resected. The patient had no other relevant medical history. She was asymptomatic and was in a good general condition. Blood tests produced normal results regarding her complete blood count, coagulation function, renal function, and liver function. Contrast-enhanced CT showed a 20-mm tumor in S3 with a variant component, which included adipose tissue (Fig. 2b). Some areas of the tumor exhibited early enhancement. Adipose tissue could also be seen on fat-suppressed magnetic resonance imaging (MRI). A metastatic liver tumor derived from the mature cystic teratoma was suspected. HAML and hepatic echinococcosis were considered as possible differential diagnoses; however, a metastatic liver tumor could not be ruled out, and laparoscopic left lateral sectionectomy was performed. The total operation time was 3 h and 1 min, and there was little intraoperative blood loss. The tumor was diagnosed as a hepatic epithelioid AML (Fig. 2c–f) after a pathological examination. The patient’s postoperative course was unremarkable, and she was discharged on the 6th day after surgery. More than 58 months have passed since the laparoscopic hepatectomy without any recurrence.

## Discussion

The present report describes 2 cases of HAMLs in which were removed by laparoscopic hepatectomy. Both HAMLs were thought to match indication for the surgical resection, because other malignant tumor, HCC for the case 1 and metastatic liver tumor for the case 2, could not be ruled out by image studies and the clinical course. The removed specimens had negative surgical margins, and there were no postoperative complications or recurrence, suggesting that laparoscopic procedures are an effective way of removing HAMLs in cases that cannot be preoperatively diagnosed correctly. At present, it is sometimes hard to diagnose HAML correctly without pathologically examining tissue specimens from the tumor. Although laparoscopic hepatectomy seems to be effective for HAMLs that are preoperatively misdiagnosed as malignant liver tumors, no previous studies have examined this clinical issue.

According to a previous review of HAMLs, preoperative diagnosis is difficult without conducting a fine-needle biopsy because of the lack of typical findings of HAML on imaging studies, including dynamic CT and EOB-MRI [15]. This lack of typical imaging findings might be due to variation in the proportions and distributions of the different tissue components of HAMLs. In case 2 the patient had previously been treated for a mature cystic teratoma, and a growing tumor with some calcification was detected at the same time. The calcification detected in the tumor by CT study in case 2 made the diagnosis more difficult, because calcification can be contained in both ovarian mature cystic teratomas [16] and HAMLs [17]. These factors made it difficult to rule out metastatic liver tumors without a pathological study of a tissue sample from the tumor, although HAML and hepatic echinococcosis were considered as possible diagnoses. Beside the fact that there is no previous report of HAML misdiagnosed as ovarian mature cystic teratoma, there are some reports of HAML misdiagnosed as HCC [8] as it was in case 1 of the present report. In case 1, the patient had normal liver function and tumor marker, AFP and PIVKA-II were within the normal ranges which can be considered to be low risk for HCC except for a dynamic CT study showing a tumor with an HCC-like enhancement pattern. In both cases, it was possible that the liver tumors were malignant; therefore, the risk of dissemination caused by a needle biopsy [18] led to the decision to completely surgically remove the tumor to obtain a therapeutic diagnosis by laparoscopic hepatectomy, a method for complete removement of the liver tumor which cannot be achieved by needle biopsy, and at the same time, a low invasive procedure for tissue sampling compared to open hepatectomy [11]. Definitive diagnoses of HAML were obtained after pathological examinations of the resected specimens in both of the present cases.

Laparoscopic hepatectomy is an effective surgical method in terms of short-term result. It is less invasive than open hepatectomy and results in shorter hospital stays and less intraoperative bleeding when performed by a surgeon who is experienced in laparoscopic hepatectomy [11]. Although most of HAMLs are considered to be benign tumors, long-term result after laparoscopic hepatectomy can’t be ignored, because some cases of HALMs with malignant behavior has been reported [4][4]. There are no reports discussing about long-term result after the laparoscopic resection for HALMs. Both 2 previously reported cases of HAMLs removed by laparoscopic procedure are followed up for less than 3 years (Table 1). Even though laparoscopic hepatectomy for HAMLs are justified if the surgical margin can be secured, since there are no reports revealing the inferiority of long-term result of laparoscopic hepatectomy compared with those of open hepatectomy for malignant liver tumors, such as HCC [12] and liver metastasis from colorectal cancer [13]. The extent of hepatectomy needed for HAMLs does not exceed to extent of hepatectomy for some other malignant liver tumors, mostly for some HCC that anatomical hepatectomy needs to be considered to improve the long-term result [14]. This means that no extra hepatectomy needs to be considered for HAML cases that were misdiagnosed as other malignant liver tumor in the preoperative study. Both cases exhibited good clinical courses after laparoscopic hepatectomy, in terms of both short-term and long-term outcomes (there were no postoperative complications in the short term, and both patients have remained disease-free for > 3 years), and no extra treatment was required.

**Table 1:** Cases of HAMLs resected by laparoscopic hepatectomy

Reference	Ref 9	Ref 10	Case 1	Case 2
Age	54	50	58	31
Sex	F	F	F	F
Preoperative diagnosis	HAML	NA	HCC	MLT
Liver function	Normal	High LFT	Normal	Normal
HBV or HCV	–	–	–	–
Preoperative observation period (m)	0	0	0	24
Tumor progression	–	–	–	+
Tumor size (cm)	3	4	1.5	2
Tumor number	1	1	1	2
Symptom	RUAP	RUAP	–	–
Hepatectomy	Hr0(1)	Hr1(L)	Hr0(S7)	Hr1(L)
Operative time (min)	NA	NA	289	181
Bleeding (ml)	NA	NA	10	little
Conversion to open surgery	–	–	–	–
Postoperative complication	–	–	–	–
Discharge (POD)	NA	12	13	5
Follow-up period (m)	12	27	37	58
Recurrence	–	–	–	–

*NA* not available, *MLT* metastatic liver tumor, *s/o* suspected, *LFT* liver function test, *RUAP* right upper abdominal pain, *POD* postoperative day

## Conclusions

In conclusion, 2 cases in which HAMLs were preoperatively diagnosed as other malignant liver tumor were successfully removed by laparoscopic hepatectomy with a correct postoperative diagnosis of HAML. Laparoscopic hepatectomy for the present 2 cases of HAML seemed to be effective for providing a correct diagnosis after the curative removement of liver tumor with a smaller invasion compared to open hepatectomy, and for denying risk of dissemination of the malignant tumor by needle biopsy that had to be considered before ruling out malignant tumor, at the same time.

## Data Availability

Not applicable.

## Abbreviations

AFP: Alpha-fetoprotein
AML: Angiomyolipoma
α-SMA: α-Smooth muscle actin
CT: Computed tomography
EOB: Ethoxybenzyl-diethylenetriamine pentaacetic acid-enhanced
HAML: Hepatic angiomyolipoma
HCC: Hepatocellular carcinoma
HMB-45: Human melanoma black 45
MRI: Magnetic resonance imaging
PIVKA-II: Protein induced by vitamin K absence or antagonist-II
SX: Segment X of the liver
